# Novel genotypes of *Hepatozoon* spp. in small mammals, Brazil

**DOI:** 10.1186/s13071-022-05216-8

**Published:** 2022-03-15

**Authors:** Bárbara C. Weck, Maria Carolina A. Serpa, Vanessa N. Ramos, Hermes R. Luz, Francisco Borges Costa, Diego G. Ramirez, Hector R. Benatti, Ubiratan Piovezan, Matias P. J. Szabó, Arlei Marcili, Felipe S. Krawczak, Sebastián Muñoz-Leal, Marcelo B. Labruna

**Affiliations:** 1 Departamento de Medicina Veterinária Preventiva E Saúde Animal, Faculdade de Medicina Veterinária E Zootecnia, Universidade de São Paulo, São Paulo, Brazil; 2 Laboratório de Ixodologia, Faculdade de Medicina Veterinária, Universidade Federal de Uberlândia, Uberlândia, MG Brazil; 3 Departamento de Patologia, Programa de Pós Graduação Em Biotecnologia Do Renorbio, Ponto Focal Maranhão, Universidade Federal Do Maranhão, São Luís, MA Brazil; 4 Departamento de Patologia, Faculdade de Medicina Veterinária, Universidade Estadual Do Maranhão, São Luís, MA Brazil; 5 Embrapa Tabuleiros Costeiros, Aracaju, SE Brazil; 6 Programa de Pós-Graduação Em Medicina E Bem-Estar Animal E Saúde Única, Universidade Santo Amaro, São Paulo, SP Brazil; 7 Setor de Medicina Veterinária Preventiva, Escola de Veterinária E Zootecnia, Universidade Federal de Goiás, Goiânia, Brazil; 8 Departamento de Ciencia Animal, Facultad de Ciencias Veterinarias, Universidad de Concepción, Chillán, Ñuble Chile

**Keywords:** Rodents, Marsupials, Hemoparasites, *Wolbachia*, Tick-borne pathogens

## Abstract

**Background:**

Small mammals (rodents and marsupials) have been poorly explored for the occurrence of apicomplexan (genus *Hepatozoon* and genera of the order Piroplasmorida) and *Anaplasmataceae* agents in Brazil. Thus, this study investigated the occurrence of *Hepatozoon* spp., Piroplasmorida, and *Anaplasmataceae* agents in small mammals in seven forest fragments in Brazil.

**Methods:**

During 2015–2018, small mammals were captured in six forest fragments in the State of São Paulo (Cerrado and Atlantic Forest biomes) and one fragment in the State of Mato Grosso do Sul (Pantanal biome). Mammal blood, liver, spleen, and lung samples were tested molecularly for the presence of DNA of *Hepatozoon,* Piroplasmorida, and *Anaplasmataceae* agents.

**Results:**

A total of 524 mammals were captured, comprising seven species of marsupials, 14 rodents, two carnivores, and one Cingulata. Four novel haplotypes (1, 2, 3, 4) of *Hepatozoon* spp. were detected in small mammals from different biomes. In São Paulo state, haplotype 1 was detected in rodents from Cerrado and a transition area of Cerrado and Atlantic Forest biomes, whereas haplotype 2 was detected in rodents from the Atlantic Forest biome. On the other hand, haplotypes 3 and 4 were restricted to rodents and marsupials, respectively, from the Pantanal biome of Mato Grosso do Sul. No host species shared more than one haplotype. Despite these distinct geographical and host associations, our phylogenetic analyses indicated that the four *Hepatozoon* haplotypes belonged to the same clade that contained nearly all haplotypes previously reported on rodents and marsupials, in addition to several reptile-associated haplotypes from different parts of the world. No mammal samples yielded detectable DNA of Piroplasmorida agents. On the other hand, the *Anaplasmataceae*-targeted polymerase chain reaction (PCR) assay amplified a sequence 100% identical to the *Wolbachia pipientis* endosymbiont of the rodent filarid *Litomosoides galizai*.

**Conclusions:**

We report a variety of *Hepatozoon* haplotypes associated with small mammals in three Brazilian biomes: Cerrado, Atlantic Forest, and Pantanal. Through phylogenetic analyses, the *Hepatozoon* agents grouped in the rodent-marsupial-reptile large clade of *Hepatozoon* spp. from the world. The detection of a *W. pipientis* associated with the rodent filarid *L. galizai* indicates that the rodent was infected by filarial nematodes.

**Graphical Abstract:**

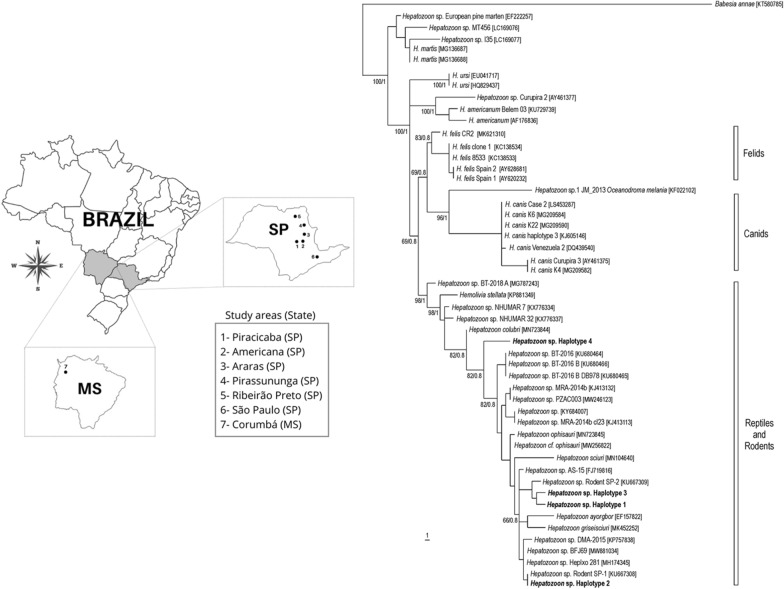

## Background

Tick-borne protozoans of the genus *Hepatozoon* and order Piroplasmorida (genera *Babesia*, *Cytauxzoon* and *Theileria*) have been associated with infections and diseases of domestic and wild mammals in Brazil [1–5]. *Hepatozoon* spp. are apicomplexan parasites characterized by a heteroxenous life-cycle, in which the intermediate hosts (vertebrates) become infected primarily through the ingestion of hematophagous arthropods (definite hosts such as ticks and mosquitoes) containing mature oocysts [6]. Alternative routes of transmission, such as the predation of infected vertebrates containing *Hepatozoon* cysts in their tissues, have been described and seem to be an important infection route for carnivore hosts [7, 8]. The order Piroplasmorida comprises tick-borne agents that infect mammalian blood cells (mostly erythrocytes), and have a major impact on farm and pet health-associated costs worldwide, but can also occur in wildlife [9, 10].

The *Anaplasmataceae* family includes bacteria of the genera *Anaplasma, Ehrlichia, Neorickettsia*, and *Wolbachia*, from which the first two encompass tick-borne agents of veterinary and public health significance world widely [11]. All genera except *Wolbachia* are known to infect vertebrate cells (mammals or birds). Through the tick bite, the bacterium enters the bloodstream and infects specific host cell types, such as neutrophils, monocytes and macrophages, platelets, erythrocytes, or endothelial cells depending on the agent [5, 12].

Small mammals such as wild rodents and marsupials are hosts of numerous species of ticks at some stage (larva, nymph, or/and adult) of their life-cycle [13]. In Brazil, several species of ticks have been found parasitizing small mammals [14–16]. Furthermore, these vertebrates have frugivorous-omnivorous habits, including the consumption of small vertebrates, invertebrates, and fruits [17]. Indeed, these habits are likely to increase the acquisition of these pathogens interspecies, especially in the case of *Hepatozoon* spp.

Various studies have reported the occurrence of apicomplexan and *Anaplasmataceae* agents in wildlife in Brazil during this century [3, 18–26], but the focus on small mammals has been much lower, which suggests that the diversity of these protozoa and bacteria might be by far underestimated, especially among marsupials. In this context, the aim of the present study was to investigate the occurrence of *Hepatozoon* spp., Piroplasmorida, and *Anaplasmataceae* agents in small mammals in seven forest fragments in Brazil.

## Methods

### Study areas and sampling procedures

**Fig. 1:**
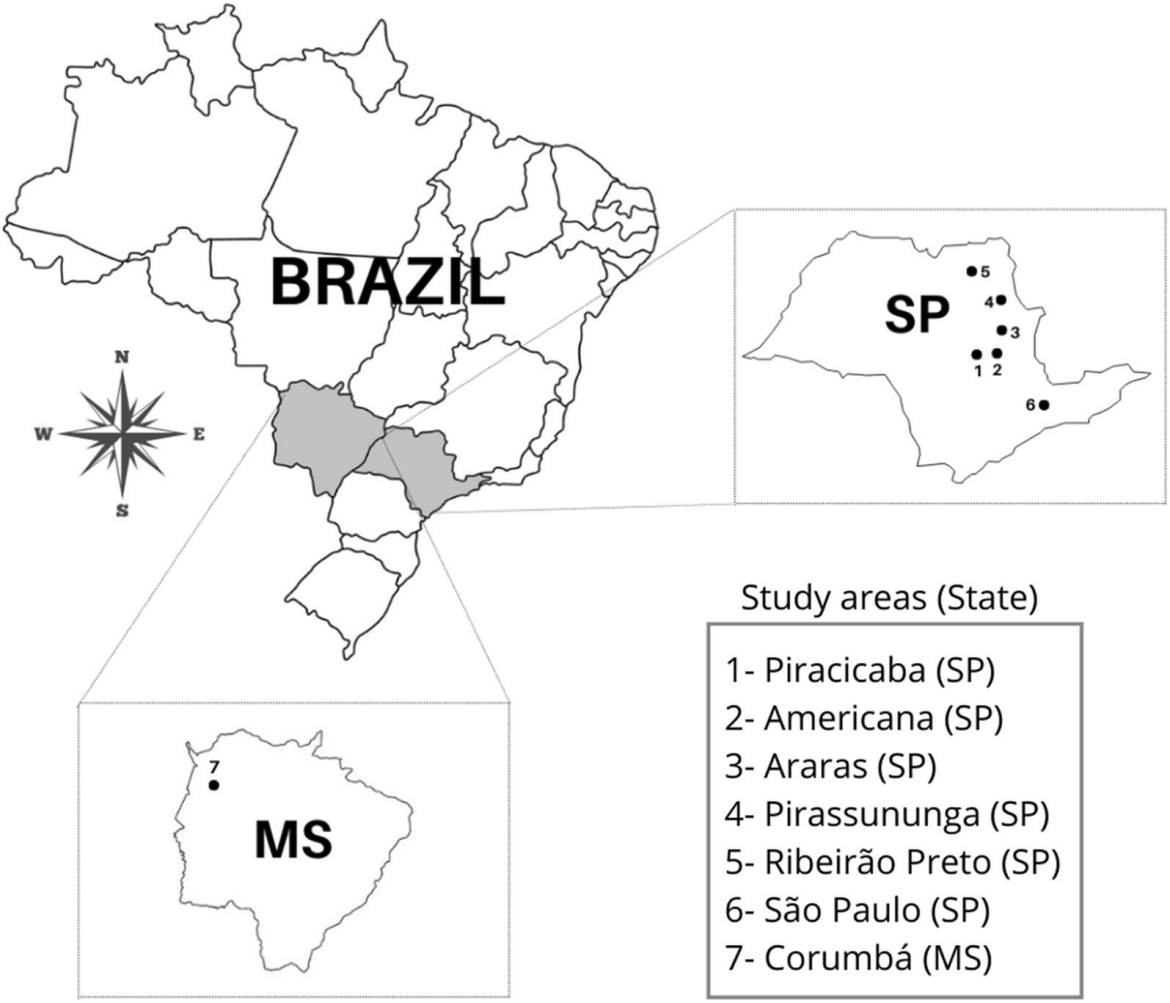
Areas in the state of São Paulo (SP) and Mato Grosso do Sul (MS) where small mammals were captured in Brazil during 2015–2018. Numbers 1 to 7 correspond to areas A1 to A7

Seven forest fragments, six of them located in the State of São Paulo and one in the State of Mato Grosso do Sul, were prospected (Fig. 1). Areas A1, A2, A3, and A4 are located in transition areas of the Cerrado and Atlantic Forest biomes; A5 belongs to the Cerrado biome; A6 is within the Atlantic Forest biome, whereas A7 is in the Pantanal biome of the state of Mato Grosso do Sul. Fieldwork was performed during the years 2015–2018 in the dry (summer) and wet (winter) seasons of each year. Small mammals were captured by Tomahawk and Sherman-like traps. General characteristics of the study sites, details on field study, and the protocols for handling the animals, anesthetic doses, storage, and identification have been published recently [16]. Briefly, blood samples were collected from all captured animals, whereas fragments of internal organs (liver, lung, and spleen) were collected from those animals that were euthanized. Collected samples were kept frozen at −20 °C until molecular analyses.

### Molecular analyses

DNA extractions from blood, liver, lung, and spleen samples were carried out using the DNeasy Tissue & Blood Kit (Qiagen, Chatsworth, CA, USA) according to the manufacturer’s instructions. In order to verify the success of extraction, an initial polymerase chain reaction (PCR) targeting the mammalian mitochondrial cytochrome b gene (*cytb*) was performed as described [27]. Subsequently, DNA samples were individually tested by a battery of PCR assays targeting protozoa of the genus *Hepatozoon,* protozoa of the order Piroplasmorida (genera *Babesia, Cytauxzoon*, and *Theileria*), and bacteria of the family *Anaplasmataceae* (Table 1)*.* Samples yielding amplicons by the initial *Hepatozoon* genus-specific PCR were submitted to PCR protocols to amplify a larger fragment of the 18S ribosomal RNA (rRNA) gene of this protozoan genus. PCR assays were performed in a total volume of 25 μl, using DreamTaq Green PCR Master Mix (Applied Biosystems, Foster City, CA, USA). Primers, PCR conditions, and positive controls are provided in Table 1. Negative controls consisted of ultrapure water. Products were resolved in 1.5% agarose gels and amplicons with the expected size purified and prepared for sequencing with the BigDye kit (Applied Biosystems, Foster City, CA, USA). An ABI PRISM 3500 Genetic Analyzer (Applied Biosystems, Foster City, CA, USA) was employed for sequencing using the same primers to perform PCRs. Obtained sequences were subjected to Basic Local Alignment Search Tool nucleotide (BLASTn) analyses to check identities with congeneric organisms available in GenBank [28].

**Table 1:** Details of the PCR primers used in the present study, with the respective positive control DNA used in the reactions

Genes	Target organisms	Primers	Sequence (5′–3′)	Amplicon size (bp)	References	Positive control in the PCR assay
18S rRNA	*Hepatozoon* spp.	HEP2 144–169 (F)	GGTAATTCTAGAGCTAATACATGAGC	574	[41]	*Hepatozoon canis*
		HEP2 743–718 (R)	ACAATAAAGTAAAAAACAYTTCAAAG			
		HAM-1(F)	GCCAGTAGTCATATGCTTGTC	1750	[42]	*H. canis*
		HPF-2(R)	GACTTCTCCTTCGTCTAAG			
		HEP-1(F)	CGCGAAATTACCCAATT^a^			
		HEP-2(R)	CAGACCGGTTACTTTYAGCAG^a^			
		HEP-4(R)	TAAGGTGCTGAAGGAGTCGTTTAT^a^			
		HPF-1(F)	CTATGCCGACTAGAGATTG^a^			
18S rRNA	Piroplasmorida	BAB2 143–167 (F)	CCGTGCTAATTGTAGGGCTAATACA	551	[41]	*Babesia vogeli*
		BAB2 694–667 (R)	GCTTGAAACACTCTARTTTTCTCAAAG			
16S rRNA	*Anaplasmataceae*	EHR 16SD (F)	GGTACCYACAGAAGAAGTCC	344	[43]	*Ehrlichia canis*
		EHR 16SD (R)	TAGCACTCATCGTTTACAG			

^a^Internal primers used only for DNA sequencing

### Phylogenetic analyses

Partial 18S rDNA sequences of *Hepatozoon* generated in the present study were aligned with Clustal X [29] and manually refined by GeneDoc [30] with corresponding *Hepatozoon* sequences available in GenBank. Two phylogenetic trees, one for a small fragment (≈550 bp) of the 18S rRNA gene (generated from the initial PCR assays), and one for a larger fragment (≈1700 bp) of the 18S rRNA gene were constructed. Both trees were inferred by using maximum parsimony (MP), as implemented in PAUP version 4.0b10 [31] with 1000 bootstrap replicates, and by Bayesian analysis (BA) using MrBayes v3.1.2 [32] with 1,000,000 replicates. The first 25% of the trees represented burn-in, and the remaining trees were used to calculate Bayesian posterior probabilities.

## Results

A total of 524 mammals were captured, comprising seven species of marsupials, 14 rodents, two carnivores, and one Cingulata (Table 2). Blood samples were collected in all cases. Among the 524 animals, 277 were euthanized and samples of liver, lung, and spleen collected. We tested a total of 1355 samples (524 blood, 277 liver, 277 lung, and 277 spleen). Expected size amplicons for *cytb* gene were obtained in all 1355 samples, thus confirming successful DNA extractions.

**Table 2:** Number of small mammals captured in the present study according to mammal order and species, study area, and type of collected samples (blood and tissue samples)

Mammals	No. captured animals / No. euthanized animals, according to areas^a^							Total (blood samples)	Total (tissue samples)
	A1	A2	A3	A4	A5	A6	A7		
Order Didelmorphia									
*Didelphis albiventris*	18/9	38/14	10/2	24/6	41/7			131	38
*Didelphis aurita*	1/1					10/2		11	3
*Gracilinanus agilis*			7/5	31/17	19/7		6/2	63	31
*Gracilinanus microtarsus*		1/1	6/2				5/4	12	7
*Marmosa constantinae*				2/2		2/2		4	4
*Monodelphis domestica*							27/11	27	11
*Philander* sp.				1/1				1	1
Order Rodentia									
*Rattus rattus*	1/1	16/14			11/10			28	25
*Mus musculus*		1/1						1	1
*Oecomys aff. marmorae*				1/1			46/13	47	14
*Nectomys squamipes*				7/4	16/10			23	14
*Necromys lasiurus*			10/9	1/1				11	10
*Oligoryzomys* spp.	8/8	1/1	18/16	28/24	23/20			78	69
*Juliomys* cf. *ossitenuis*				2/2				2	2
*Akodon* sp.				6/5	3/2	6/6		15	13
*Hylaeamys megacephalus*				6/3	1/1			7	4
*Euryoryzomys russatus*			4/3			9/9		13	12
*Cavia* sp.			2/2	1/1				3	3
*Clyomis laticeps*							2/1	2	1
*Thrichomys pachyurus*							34/6	34	6
*Cerradomys* sp.				2/1				2	1
Order Cingulata									
*Dasypus novemcinctus*	1/1							1	1
Order Carnivora									
*Galictis cuja*			1/0					1	
*Nasua nasua*	1/1						2/1	3	2
Not identified (NI)									
NI			3/3	1/1				4	4
Total	30	57	61	113	114	27	122	524	277

^a^Areas A1 to A7 are indicated in Fig. 1

Among the 524 sampled animals, 21 (4.0%) yielded amplicons in the initial *Hepatozoon* genus-specific PCR targeting a 574-bp fragment of the 18S rRNA gene*.* These 21 animals comprised 11 individual rodents from the state of São Paulo (one from A4, one from A5, and nine from A6), and six individual marsupials and four rodents from the state of Mato Grosso do Sul (A7) (Table 3). Only one of the 21 *Hepatozoon*-infected animals yielded amplicons from blood. The remaining 20 infected mammals yielded amplicons from the lung only (7 animals), liver only (3), spleen only (1), lung and spleen (4), and lung, liver, and spleen (5). Amplicons from the 21 PCR-positive small mammals were submitted to DNA sequencing, resulting in four 18S rRNA new haplotypes (1, 2, 3, and 4) of the genus *Hepatozoon*. Haplotypes 1 and 2 were restricted to rodents of the state of São Paulo, whereas the other two haplotypes were found only in the state of Mato Grosso do Sul, being haplotype 3 on rodents, and haplotype 4 on marsupials (Table 3).

**Table 3:** Data of the 21 small mammals that were shown to contain *Hepatozoon* DNA by two PCR protocols, one targeting a small fragment (≈540 bp) and the other targeting a large fragment (≈1700 bp) of the *Hepatozoon* 18S RNA gene

Mammal individual Code	Area^a^	Mammal species	Results of PCR targeting the 18S rRNA gene		
			Positive samples	Small fragment haplotype^b^	Large fragment haplotype^c^ [GenBank accession number]
PR065	A4	*Nectomys squamipes*	Spleen	1^d^	
RP033	A5	*Oligoryzomys* sp.	Lung	1	
HF006	A6	*Akodon* sp.	Lung, liver, spleen	2	
HF010	A6	*Akodon* sp.	Liver	2	
HF015	A6	*Akodon* sp.	Liver	2	HF015 [OM033663]
HF025	A6	*Akodon* sp.	Blood	2	
HF027	A6	*Akodon* sp.	Lung	2	
HF005	A6	*Akodon* sp.	Lung, liver, spleen	2	HF005 [OM033660]
HF009	A6	*Euryoryzomys russatus*	Lung	2	HF009 [OM033661]
HF014	A6	*E. russatus*	Lung	2	HF014 [OM033662]
HF021	A6	*E. russatus*	Lung	2	
PS003	A7	*Gracilinanus microtarsus*	Liver	4	
PS001	A7	*Monodelphis domestica*	Lung, spleen	4	PS001 [OM033664]
PS063	A7	*M. domestica*	Lung, liver, spleen	4	
PS082	A7	*M. domestica*	Liver, spleen	4	
PS113	A7	*M. domestica*	Lung, liver, spleen	4	
PS130	A7	*M. domestica*	Lung, spleen	4	
PS085	A7	*Oecomys marmorae*	Lung	3	PS085 [OM033665]
PS119	A7	*O. marmorae*	Lung, spleen	3	
PS125	A7	*O. marmorae*	Lung, liver, spleen	3	
PS022	A7	*Thrichomys pachyurus*	Lung	3	

^a^Areas A4, A5, A6, and A7 are indicated in Fig. 1;

^b^see Fig. 2;

^c^see Fig. 3;

^d^GenBank accession number for haplotype 1 is OM033659; haplotypes 2, 3, and 4 are within the respective large fragment haplotypes, for which GenBank accession numbers are provided in the last column of the Table

Considering the closest identities under 100% query-cover through BLAST analysis, the *Hepatozoon*-haplotype 1, detected in the rodents *Nectomys squamipes* and *Oligoryzomys nigripes,* was 98.7% (532/539 bp) identical to *Hepatozoon* sp. detected in the tick *Ixodes* sp. from Chile (MH174345) and *Hepatozoon* sp. detected in the rodent *Phyllotis darwini* from Chile (MW881034). The *Hepatozoon*-haplotype 2, detected in the rodents *Akodon* sp. and *Euryoryzomys russatus,* was 99.8% (543/544 bp) identical to the same two *Hepatozoon* sp. sequences from Chile (MH174345, MW881034). Similarly, the *Hepatozoon*-haplotype 3, detected in the rodents *Oecomys mamorae* and *Thrichomys pachyurus,* was 98.2% (532/542 bp) identical to the same two *Hepatozoon* sp. sequences from Chile (MH174345, MW881034). Finally, the *Hepatozoon*-haplotype 4, detected in the marsupials *Gracilinanus microtarsus* and *Monodelphis domestica,* was 98.0% (532/543 bp) identical to *Hepatozoon* sp. detected in the reptile *Caiman crocodilus* from Brazil (KJ413132, KY684007).

Based on the above samples that generated four distinct haplotypes, we attempted to generate a larger sequence (≈1700 bp) of the *Hepatozoon* 18S rRNA gene from at least one small mammal of each haplotype. Although no larger fragment was generated from haplotype 1-representatives, we did generate it from four haplotype 2-representatives, from which their identical 18S rRNA sequences, here designated as *Hepatozoon* sp. HF005, HF009, HF014, and HF015, were by BLAST analysis 99.2% (1715/1729 bp) identical to *Hepatozoon ayorgbor* from the snake *Python regius* from Ghana (EF157822) (Table 3). A larger 18S rDNA fragment of a haplotype 3-representative (designated as *Hepatozoon* sp. PS085) was 98.2% (1710/1741 bp) identical to the same *H. ayorgbor* sequence mentioned above (EF157822). Similarly, from a haplotype 4-representative (designated as *Hepatozoon* sp. PS001), its larger 18S rDNA fragment was 98.2% (1692/1723 bp) identical to *H. ayorgbor* (EF157822) (Table 3).

**Fig. 2:**
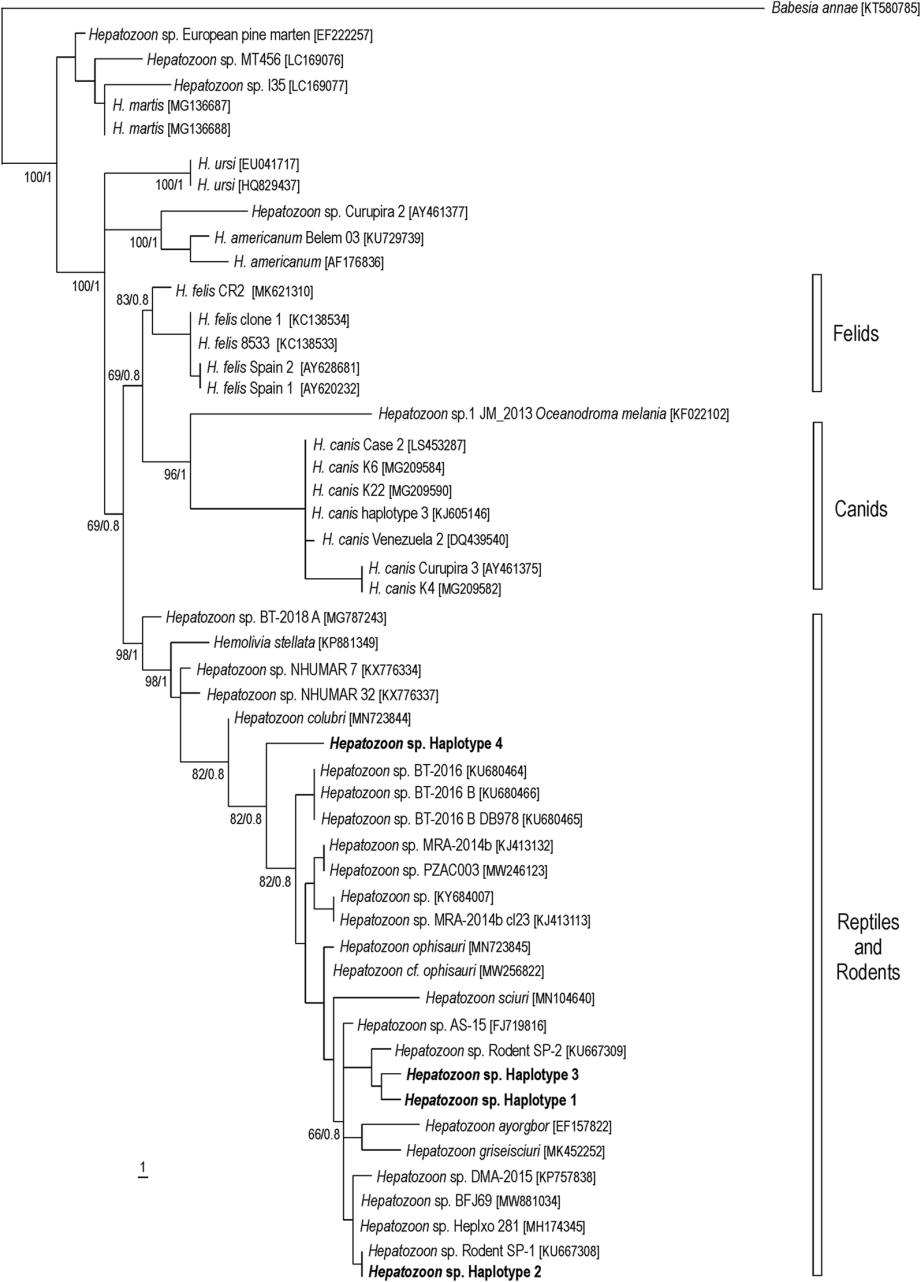
Maximum parsimony and Bayesian tree inferred from 18S rRNA gene partial sequences (566 characters, 77 informative sites) of *Hepatozoon* spp., with *Babesia annae* as outgroup. Numbers at nodes are the support values for the major branches (bootstrap/posterior probability). The sequences obtained in this study are in bold. Numbers in brackets correspond to GenBank accession numbers

**Fig. 3:**
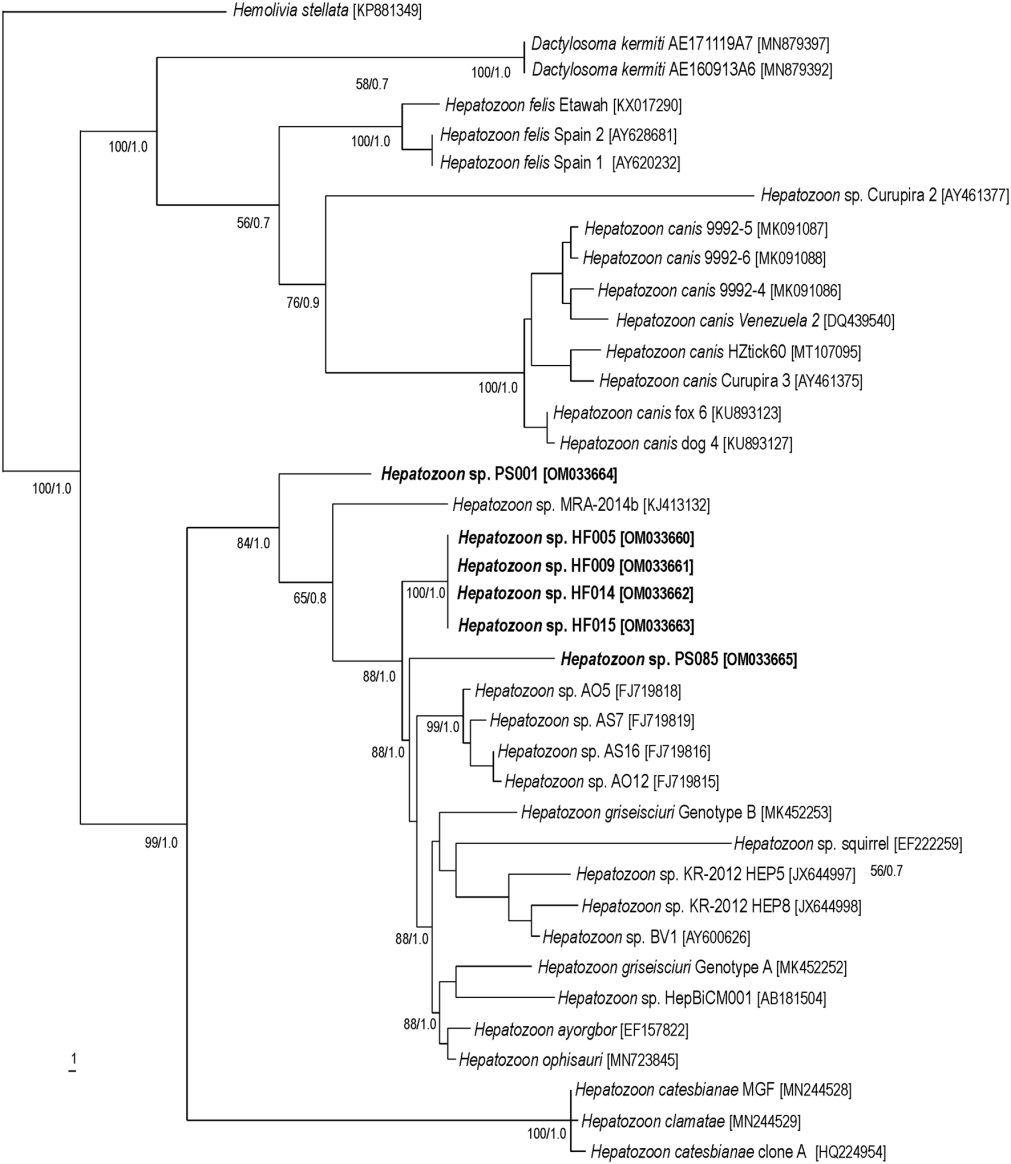
Maximum parsimony and Bayesian tree inferred from 18S rRNA gene partial sequences (1676 characters, 113 informative sites) of *Hepatozoon* spp., with *Hemolivia stellata* as outgroup. Numbers at nodes are the support values for the major branches (bootstrap/posterior probability). The sequences obtained in this study are in bold. Numbers in brackets correspond to GenBank accession numbers

In the phylogenetic tree inferred from short partial sequences (≈540 bp) of the 18S rRNA gene of *Hepatozoon* spp., haplotypes 1, 2, 3, and 4 branched within a large clade composed by many *Hepatozoon* haplotypes associated with rodents and reptiles from different parts of the world. This large clade was sister to another large clade that contained *Hepatozoon* haplotypes associated with canids (*Hepatozoon canis*) and felids (*Hepatozoon felis*) under 66 (MP) and 0.8 (BA) bootstrap supports (Fig. 2). This topology was somewhat similar in the phylogenetic tree inferred from long partial sequences (≈1700 bp) of the 18S rRNA gene of *Hepatozoon* spp., in which the sequences of the present study branched within a large clade containing many haplotypes associated with rodents and a few reptiles under 84 (MP) and 1.0 (BA) bootstrap supports (Fig. 3).

None of the 1355 samples yielded amplicons by the Piroplasmorida-PCR protocol. One blood sample from an *Oligoryzomys* sp. from Piracicaba (A1) yielded amplicons by the *Anaplasmataceae-*PCR protocol. These amplicons generated a DNA sequence 100% (306/306 bp) identical to the *Wolbachia pipientis* endosymbiont of the rodent filarid *Litomosoides galizai* (AJ548800).

## Discussion

In the present study, four different haplotypes (1, 2, 3, 4) of *Hepatozoon* spp. were detected in small mammals from different biomes among two Brazilian states, São Paulo and Mato Grosso do Sul. In São Paulo state, haplotype 1 was detected in rodents from Cerrado and a transition area of Cerrado and Atlantic Forest biomes, whereas haplotype 2 was detected in rodents from the Atlantic Forest biome. On the other hand, haplotypes 3 and 4 were restricted to rodents and marsupials, respectively, from the Pantanal biome of Mato Grosso do Sul. No host species shared more than one haplotype. Despite these distinct geographical and host associations, our phylogenetic analyses indicated that the four *Hepatozoon* haplotypes belonged to the same clade that contained nearly all haplotypes previously reported on rodents and marsupials, in addition to several reptile-associated haplotypes from different parts of the world. This general topology was confirmed by our analysis inferred from nearly complete sequences (≈1700 bp) of the 18S rRNA gene of *Hepatozoon* spp.

Previous molecular detections of *Hepatozoon* spp. on small rodents and marsupials in Brazil are restricted to six studies [20, 25, 33–36]. Indeed, the vast majority of these records were on small rodents (at least 12 rodent species), in contrast to only two records on marsupials: *Hepatozoon* sp. PCR165 on one *Thylamys macrurus* [35], and *H. canis* on two *Didelphis albiventris* [34]*.* Except for this later record of *H. canis,* all previous records on small rodents and marsupials in Brazil demonstrated that the *Hepatozoon* spp. haplotypes belonged to the large clade composed of haplotypes associated with rodents, marsupials, and reptiles, in agreement with the results of the present study. Unfortunately, many of the sequences from previous studies were from regions of the 18S rRNA gene different from the present study (in the case of our Fig. 2) or much shorter than 1700 bp (in the case of our Fig. 3); for this reason, they were not included in our phylogenetic analysis.

We retrieved rodent-associated-*Hepatozoon* haplotypes (only those > 500 nucleotides) from previous Brazilian studies [20, 25, 33–36] from GenBank and aligned them with the 18S rRNA long haplotypes (≈1700-haplotypes) of the present study (data not shown), in order to determine whether any of them matched 100% to any part of the long haplotypes. As shown in Table 4, haplotypes HF005, HF009, HF014, and HF015 (all from the rodents *Akodon* sp. and *E. russatus* of the state of São Paulo) had internal regions that were 100% identical to 516–1007-bp haplotypes of *Hepatozoon* sp., previously reported on the rodents *Akodon montensis*, *Akodon cursor*, and *Galea spixii* from three Brazilian states, including São Paulo [25, 33]. Haplotype PS085 (from the rodent *O. mamorae* from the Pantanal biome of Mato Grosso do Sul) had regions that were 100% identical to 573–625–bp haplotypes of *Hepatozoon* sp., previously reported on this same rodent species and the same Brazilian biome [35], indicating that PS085 might be primarily related to *O. mamorae* in the Pantanal. On the other hand, no *Hepatozoon* sequence > 500 bp from GenBank matched 100% to haplotype PS001, which was detected in the marsupial *M. domestica* in the Pantanal biome. This result points PS001 as a novel *Hepatozoon* haplotype associated with marsupials in the Pantanal biome of Mato Grosso do Sul state, where other closely related haplotypes seem to be associated primarily with rodents and reptiles.

**Table 4:** List of short haplotypes (at least 500 bp long) previously reported in Brazil that were 100% identical to parts of the 18S rRNA long haplotypes (≈1700-haplotypes) of the present study

Long haplotypes of the present study (Brazilian state)	*Hepatozoon* short haplotypes from GenBank that were 100% identical to regions of the long ≈1700 bp *Hepatozoon* haplotypes of the present study			
	GenBank accession number (No. nucleotides)^a^	Host	Brazilian state	References
HF005, HF009, HF014, and HF015 (São Paulo)	KU667308 (1007)	*Akodon montensis*	São Paulo	[33]
	MH111405 (517)	*Akodon cursor*	Rio de Janeiro	[25]
	MH111407 (528)	*Galea spixii*	Bahia	[25]
	MH111408 (564)	*A. montensis*	Rio de Janeiro	[25]
	MH111409 (516)	*A. cursor*	Rio de Janeiro	[25]
	MH111410 (535)	*A. montensis*	Rio de Janeiro	[25]
	MH111417 (520)	*A. montensis*	São Paulo	[25]
PS085 (Mato Grosso do Sul)	KX776336 (625)	*Oecomys mamorae*	Mato Grosso do Sul	[35]
	KX776347 (575)	*O. mamorae*	Mato Grosso do Sul	[35]
	KX776348 (607)	*O. mamorae*	Mato Grosso do Sul	[35]
	KX776353 (573)	*O. mamorae*	Mato Grosso do Sul	[35]
PS001 (Mato Grosso do Sul)	None			

^a^None of these sequences was included in the phylogenetic trees of the present study because they were from regions of the 18S rRNA gene different from the alignment used in Fig. 2 or they were much shorter than the ≈1700-bp alignment of Fig. 3

Vertebrate hosts acquire *Hepatozoon* infection through the ingestion of a hematophagous arthropod containing mature oocysts. Alternatively, infection can be acquired by intrauterine transmission or by predation of infected vertebrates containing merozoites (intermediate hosts) or cystozoites (paratenic host) [37]. Previous studies with wild rodents have detected *Hepatozoon* meronts in the liver or lungs (reviewed by [33]). Regarding the infection by *Hepatozoon milleri* in the rodent *A. montensis* in Brazil, Demoner et al. [33] detected meronts only in the liver, whereas cystozoites were detected in the spleen, lungs, and kidneys. Since only one of the 21 infected hosts yielded amplicon in the blood (i.e., gamont detection), we infer that the small mammals of the present study contained meronts and/or cystozoites, hence, they might be acting as intermediate or paratenic hosts to *Hepatozoon* spp. in the studied areas. As previously stated [35, 36, 38], rodents might play a role in the epidemiological cycle of reptile-associated *Hepatozoon* spp. rather than the Carnivora-associated species (e.g., *H. canis*, *H. felis*), as indicated by ours and previous phylogenetic analyses.

Among the seven sampled areas of the present study, *Hepatozoon* spp. infection was detected only in areas A4, A5, A6, and A7. According to previous analyses [16], these four areas presented significantly higher diversity of small mammals than areas A1, A2, and A3. Higher biodiversity could favor the transmission of *Hepatozoon* by interspecies predation, a likely important transmission route of this protozoan [35]. In addition, higher diversity of ticks could also facilitate *Hepatozoon* transmission*.* Again, it is noteworthy that areas A4, A5, A6, and A7 presented higher diversity of ticks than the remaining areas [16], suggesting that the life-cycle of small mammal-associated *Hepatozoon* spp. is under a complex interaction of vertebrate and invertebrate hosts.

Regarding the other PCR assays of the present study, none of the samples yielded detectable DNA to Piroplasmorida agents. This result contrasts with previous studies that reported different Piroplasmorida haplotypes among small mammals from other regions of Brazil, including studies that employed the same PCR protocol of the present study [3, 20, 24, 39]. Therefore, these contrasting results could be related to uneven distribution of Piroplasmorida agents among the populations of small mammals along different geographical regions of Brazil, due to reasons yet to be determined.

The PCR assay targeting *Anaplasmataceae* bacteria yielded amplicons from the blood of an *Oligoryzomys* sp.: a 16S rRNA partial sequence 100% identical to *W. pipientis* associated with the rodent filarid *L. galizai*. This is indirect evidence that this rodent was infected by filarial nematodes. In previous studies, similar PCR assays amplified *Wolbachia* fragments from wild mammals (monkeys and opossums) and domestic dogs, leading the authors to conclude that these animals were infected by filarial parasites [22, 40].

## Conclusions

We report a variety of *Hepatozoon* haplotypes associated with small mammals in three Brazilian biomes: Cerrado, Atlantic Forest, and Pantanal. Through phylogenetic analyses, the *Hepatozoon* agents grouped in the rodent-marsupial-reptile large clade of *Hepatozoon* spp. from the world. Our phylogenetic analyses suggest that small mammals might play a role in the epidemiology cycle or reptile-associated *Hepatozoon* spp. rather than the Carnivora-associated *Hepatozoon* species. Since the *Hepatozoon* detections were restricted to areas with higher diversity of small mammals and ticks, this condition could facilitate *Hepatozoon* transmission*.* Finally, the detection of a *W. pipientis* associated with the rodent filarid *L. galizai* indicates that the rodent was infected by filarial nematodes.

## Data Availability

The datasets supporting the findings of this article are included or indicated within the article. The nucleotide sequences were deposited in GenBank under the accession numbers OM033659 to OM033665 for the *Hepatozoon* spp. haplotypes, and OM044588 for *Wolbachia* sp.
